# Hookworm-Related Anaemia among Pregnant Women: A Systematic Review

**DOI:** 10.1371/journal.pntd.0000291

**Published:** 2008-09-17

**Authors:** Simon Brooker, Peter J. Hotez, Donald A. P. Bundy

**Affiliations:** 1 London School of Hygiene and Tropical Medicine, London, United Kingdom; 2 Malaria Public Health and Epidemiology Group, Centre for Geographic Medicine, KEMRI/Wellcome Trust Research Laboratories, Nairobi, Kenya; 3 Department of Microbiology, Immunology, and Tropical Medicine, The George Washington University Medical Center, Washington, D.C., United States of America; 4 Human Hookworm Vaccine Initiative, Sabin Vaccine Institute, Washington, D.C., United States of America; 5 Human Development Division, The World Bank, Washington, D.C., United States of America; The University of Queensland, Australia

## Abstract

**Background and Objectives:**

Hookworm infection is among the major causes of anaemia in poor communities, but its importance in causing maternal anaemia is poorly understood, and this has hampered effective lobbying for the inclusion of anthelmintic treatment in maternal health packages. We sought to review existing evidence on the role of hookworm as a risk factor for anaemia among pregnant women. We also estimate the number of hookworm infections in pregnant women in sub-Saharan Africa (SSA).

**Methods:**

Structured searches using MEDLINE and EMBASE as well as manual searches of reference lists were conducted, and unpublished data were obtained by contacting authors. Papers were independently reviewed by two authors, and relevant data were extracted. We compared haemoglobin concentration (Hb) according to intensity of hookworm infection and calculated standardised mean differences and 95% confidence intervals. To estimate the number of pregnant women, we used population surfaces and a spatial model of hookworm prevalence.

**Findings:**

One hundred and five reports were screened and 19 were eligible for inclusion: 13 cross-sectional studies, 2 randomised controlled trials, 2 non-randomised treatment trials and 2 observational studies. Comparing uninfected women and women lightly (1–1,999 eggs/gram [epg]) infected with hookworm, the standardised mean difference (SMD) was −0.24 (95% CI: −0.36 to −0.13). The SMD between women heavily (4000+ epg) infected and those lightly infected was −0.57 (95% CI: −0.87 to −0.26). All identified intervention studies showed a benefit of deworming for maternal or child health, but since a variety of outcomes measures were employed, quantitative evaluation was not possible. We estimate that 37.7 million women of reproductive age in SSA are infected with hookworm in 2005 and that approximately 6.9 million pregnant women are infected.

**Conclusions:**

Evidence indicates that increasing hookworm infection intensity is associated with lower haemoglobin levels in pregnant women in poor countries. There are insufficient data to quantify the benefits of deworming, and further studies are warranted. Given that between a quarter and a third of pregnant women in SSA are infected with hookworm and at risk of preventable hookworm-related anaemia, efforts should be made to increase the coverage of anthelmintic treatment among pregnant women.

## Author Summary

Anaemia affects large numbers of pregnant women in developing countries and increases their risk of dying during pregnancy and delivering low birth weight babies, who in turn are at increased risk of dying. Human hookworm infection has long been recognized among the major causes of anaemia in poor communities, but understanding of the benefits of the management of hookworm infection in pregnancy has lagged behind the other major causes of maternal anaemia. Low coverage of anthelmintic treatment in maternal health programmes in many countries has been the result. After systematically reviewing the available literature we observed that increasing hookworm infection intensity is associated with lower haemoglobin levels in pregnant women. We also estimate that between a quarter and a third of pregnant women in sub-Saharan Africa are infected with hookworm and at risk of preventable hookworm-related anaemia. However, all identified intervention studies showed a benefit of deworming for maternal or child health and we argue that increased efforts should be made to increase the coverage of anthelmintic treatment among pregnant women.

## Introduction

Anaemia is a major factor in women's health, especially reproductive health in developing countries. Severe anaemia during pregnancy is an important contributor to maternal mortality [1], as well as to the low birth weight which is in turn an important risk factor for infant mortality [2]–[3]. Even moderate anaemia makes women less able to work and care for their children [4]. The causes of anaemia are multi-factorial, including diet, infection and genetics, and for some of the commonest causes of anaemia there is good evidence of the effectiveness of simple interventions: for example, iron supplementation [5], long-lasting insecticide nets and intermittent preventive treatment for malaria [6]–[7].

Hookworm infection has long been recognized among the major causes of anaemia in poor communities [8], but understanding of the benefits of the management of hookworm infection in pregnancy has lagged behind the other major causes of maternal anaemia. An epidemiological study in 1995 highlighted the paradox presented to public health workers that an estimated one-third of all pregnant women in developing countries were infected with hookworm and yet, in the absence of safety data, the appropriate advice then current was to avoid the use of anthelmintics in pregnancy [9]. Furthermore, the lack of an acceptable intervention constrained the development of evidence-based understanding of the impact of hookworm infection on maternal anaemia [10]. These issues were addressed directly by de Silva and colleagues [11], who analysed the safety profile of some 20 years of mebendazole use in antenatal clinics in Sri Lanka. In 2002, WHO published new guidance indicating that pregnant women should be treated for hookworm infection, ideally after the first trimester [12]. This immediately provided the opportunity for improved service delivery, and also encouraged studies to assess the contribution of hookworm to anaemia in pregnancy and the impact of treatment, some of which have been undertaken since 2002. These provide a rich new source of data to help inform public health decision making, and in this paper we present a systematic review of hookworm as a risk factor for anaemia among pregnant women. We also estimate the extent of the problem of hookworm among pregnant women living in sub-Saharan Africa, where hookworm remains an intractable reproductive health problem.

## Methods

### Data sources and search strategy

A systematic search of published articles was undertaken in July 2007 and repeated again in October 2007. The online databases MEDLINE (1970–2007) and EMBASE (1980–2007) were used to identify relevant studies, using the Medical Subject Headings (MSHs) *pregnancy* or *pregnant* AND *hookworm*, *Necator americanus*, *Ancylostoma duodenale*, *intestinal parasites*, *geohelminths* or *soil-transmitted helminths* AND *an(a)emia*, *h(a)emoglobin* or *h(a)ematocrit*. All permutations of MSHs were entered and each search was conducted twice to ensure accuracy. The search did not exclude non-English language papers. The abstracts of returned articles were then reviewed, and if they did not explicitly investigate the association between hookworm and anaemia, they were discarded. Potentially useful articles were retrieved. We also reviewed reference lists of identified articles and hand searched reviews. Where suitable papers did not provide information in a relevant format, authors were emailed and requested to provide relevant summaries of data. SB undertook the literature search and scanned the results for potentially relevant studies, retrieved the full article, and contacted authors. SB and PJH independently assessed every relevant paper, with no disagreements arising, and SB used a pre-formed database to abstract information.

We followed the reporting checklist of the Meta-analysis of observational studies in epidemiology (MOOSE) group [13]. The primary outcome analysis was haemoglobin concentration (Hb), and our hypothesis was that haemoglobin concentration is associated with the intensity of hookworm infection. Data without quantitative measures of Hb and hookworm infection intensity were excluded. No distinction could be made between the two different hookworm species, *Necator americanus* and *Ancylostoma duodenale*, because none of the studies used specific methods to differentiate the species, and routine coprology is unable to do this. Studies had to be based on at least 30 individuals. No scoring of quality of studies was undertaken. However, a description of statistical methods employed, including whether adjustment for potentially confounding variables, is provided. For randomised controlled trials, information is provided on key components of study design as recommended by the CONSORT statement [14].

### Data analysis

Data were stratified according to the intensity of infection, based on thresholds recommended by WHO: light (1–1,999 epg); moderate (2,000–3,999 epg); and heavy (4000+ epg). Estimates of Hb were assessed for each intensity category and differences between categories were presented as a standardized mean difference (SMD) and 95% confidence interval. These were calculated with a random-effects model according to the DerSimonian and Laird method [15]. Heterogeneity was assessed by the I^2^ test with values greater than 50% representing significant heterogeneity. When heterogeneity between studies was found to be significant, pooled estimates were based on random-effect models and the Hedges method of pooling. Results were displayed visually in forest plots. Bias was investigated by construction of funnel plots and by the statistical tests developed by Begg & Mazumdar [16] and Egger et al. [17]. Analysis was performed using the ‘metan’ and related functions in STATA version 10 (College Station, TX).

### Estimating population at-risk of hookworm-related anaemia

We attempted to estimate the number of pregnant women infected with hookworm in hookworm-endemic countries in sub-Saharan Africa. To estimate the number of pregnant women, we used population data from the Gridded Population of the World (GPW) version 3.0 β [18]. GPW3.0β is a global human population distribution map derived from areal weighting of census data from 364,111 administrative units to a 2.5′×2.5′ spatial resolution grid. Country-specific medium variant population growth rates and proportions of the female population aged 15–49 years available from the United Nations Population Division – World Population Prospects [UNPD-WPP] database [19] were used to project this age cohort of the population total to 2005 using ArcView (Environmental Systems Research Institute Inc., CA, USA). The number of pregnant women was estimated separately for each country from the crude birth rate (number of births over a given period divided by the person-years lived by the population over that period); this will be an under-estimate as it does not include women experiencing miscarriages and stillbirths, which are not routinely reported. Hookworm prevalence was defined on the basis of an existing model which uses satellite-derived climatic factors to predict the geographical distribution and prevalence of hookworm among school-aged children [20]. In the absence of relevant empirical data, we assume that infection prevalence is equivalent in school-aged populations and pregnant women; this is probably an under-estimate since hookworm prevalence is generally higher in adult populations [21]. We also assume that no large-scale hookworm control has been undertaken. Extractions of population at risk by prevalence of hookworm were then conducted in ArcView 3.2.

## Results

Our literature searches identified 105 citations and from this list 30 potentially relevant research studies were identified; the remaining citations were either research studies among non-pregnant women, reviews or editorials. Of these 30 potentially relevant studies, 19 were determined to be eligible, including 13 cross-sectional studies, 2 randomised controlled trials, 2 non-randomised treatment trials and 2 observational studies.

### Association between hookworm infection and haemoglobin

**Figure 1:**
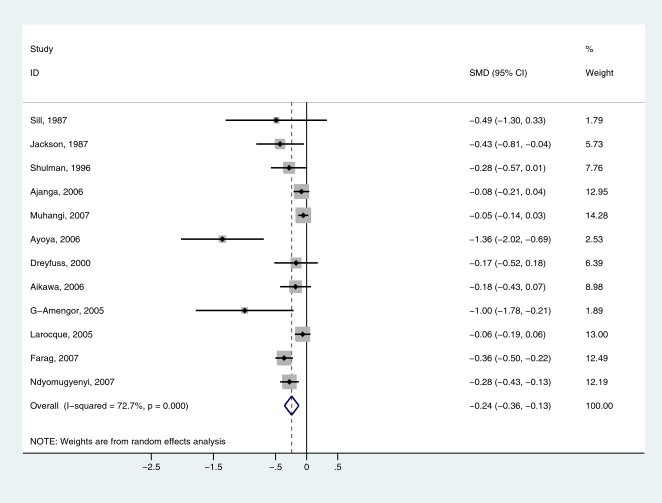
Forest plot of the difference in haemoglobin (Hb) concentration among pregnant women uninfected with hookworm and women harbouring a light (1–1,999 eggs/gram) hookworm infection. Standardised mean difference less than zero indicate lower Hb levels in lightly infected women compared to uninfected women. The diamond represents the overall pooled estimates of the effect of light hookworm infection on Hb.

**Figure 2:**
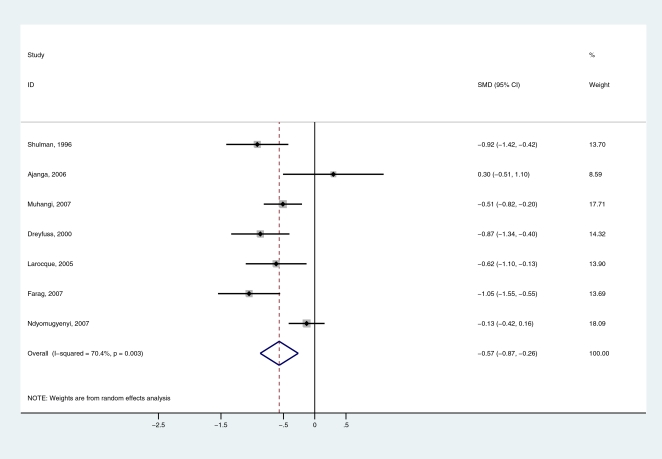
Forest plot of the difference in haemoglobin (Hb) concentration among pregnant women women harbouring a light (1–1,999 eggs/gram) hookworm infection and women harbouring a heavy (4,000+ eggs/gram) infection. Standardised mean difference less than zero indicate lower Hb levels in heavily infected women compared to lightly infected women. The diamond represents the overall pooled estimates of the effect of heavy hookworm infection on Hb.

13 studies presented observational data on the relationship between hookworm infection and haemoglobin concentration: eight from Africa, three from Asia and two from Latin America. The characteristics of the cross-sectional studies included are presented in Table 1. The data were stratified according to the intensity of infection. In four of the studies, none of the woman included had an intensity of infection that exceeded 2,000 epg; in eight studies women had an infection intensity that exceeded 4,000 epg. Comparing uninfected women and women lightly (1–1,999 epg) infected with hookworm, the standardized mean difference (SMD) in Hb was −0.72 (95% CI: −1.26 to −0.18) (n = 13), indicating that even women lightly infected with hookworm have lower Hb levels than uninfected women. However, there was variation in the differences observed and examination of forest plots suggested heterogeneity of effect, which was statistically significant (I^2^ score of 72.9%). This was explained by inclusion of the study by Rodríguez-Morales et al. [22] which collated data from nine states across Venezuela. Omitting this study from the analysis, the SMD between women uninfected and lightly infected was −0.24 (95% CI: −0.36 to −0.13) (Figure 1). Omission of other studies made little or no difference to the overall effect. There was slight evidence of bias using the Egger test (p = 0.008) and the Begg test (p = 0.07): the relatively small study by Ayoya et al. [23] in Mali showed evidence of effects that differed from the larger studies. Heavy hookworm infection was also significantly associated with a lower Hb level compared to light infection: the standardized mean difference in Hb was −0.57 (95% CI: −0.87 to −0.26) (n = 7) (Figure 2). No evidence of bias was observed.

**Table 1:** The impact of hookworm infection on haemoglobin concentration in pregnant women.

Setting	Participants and year of study	Prevalence of parasites (%)^a^	Prevalence of anaemia (threshold used)	Statistical methods and potential confounders adjusted for^a^	Study
Liberia	128 women attending antenatal clinic aged 14–43 y, 88% in 1^st^ or 2^nd^ trimester, 1985	Hw = 30.0	78% (<110 g/L)	Unadjusted t-test.	[50]
Kilifi, Kenya	251 women attending antenatal clinic aged 15–41 y, 88% in 1^st^–3rd trimester, 1993	Hw = 74.9Pf = 23.6	75.6% (<110 g/L)	Unadjusted Kruskal-Wallis test	[51]
Ashanti Region, Ghana	205 women attending antenatal clinic aged 15–49 y, who were healthy, in 3^rd^ trimester and resident in the area, 2003–2005	Hw = 8.1Pf = 35.1	57.1% (<100 g/L)	Unadjusted t-test.	[52]
Bamako, Mail	131 randomly selected women attending antenatal clinic aged 18–45 y, who were healthy, in 1^st^–3^rd^ trimester and resident in the area, 2002	Hw = 8Pf = 11	47% (<110 g/L)	Multivariate logistic regression: age, gestation, trimester, SES, abnormal vaginal discharge, food constraints, Pf & Sh.	[23]
Ukerewe Island, Tanzania	972 women attending antenatal clinics aged 15–45 y, who were healthy and resident in the area, 2004	Hw = 56.3Pf = 16.4Sm = 63.5	66.4% (<110 g/L)	Multivariate logistic regression: age, trimester, Pf & Sm.	[53]
Entebbe, Uganda	2507 women attending antenatal clinic aged 14–47 y, who were healthy and resident in the area, 2003–2005	Hw = 44.5Pf = 10.9	39.7% (<112 g/L)	Multivariate logistic regression: age, SES & gravidity.	[54]
Masindi, Uganda	802 women attending first antenatal visit aged 14–42 y, who were healthy and resident in the area,2003–4	Hw = 66.6Pf = 35.2	20.8% % (<100 g/L)	Multivariate logistic regression: age, gravidity, gestation, iron deficiency, Pf, Sm, Al &Tt.	[55]
Pemba Island, Tanzania	857 women attending antenatal clinic aged 15–49 y, who were healthy, in 3^rd^ trimester and resident in the area, 2004	Hw = 32.9*Pf* = 7.4	Severe anaemia: 5.5% (<70 g/L)	Multivariate logistic regression: age, gestation, gravidity, SES, dietary intake, iron deficiency, Hp & Pf.	[56]
Iquitos, Peru	1042 women attending antenatal clinics aged 18–42 y living in rural and peri-urban areas, 2003–4	Hw = 47.2Tt = 82.3Al = 63.9	47.3% (<110 g/L)	Multivariate logistic regression: age, SES, education, gestation, Al & Tt.	[57]
Nine states, Venezuela	1038 women attending antenatal clinics in rural and peri-urban areas, 2003–4	Hw = 8.1	65.1% (<120 g/L)	Relative risks adjusted for Al, Tt & other protozoan infections.	[22]
Papua New Guinea	30 women attending antenatal clinic for first time, 1985	Hw = 60Pf/Pv = 7	44% (<100 g/L)	Unadjusted t-test.	[58]
Sarlahi district, Nepal	334 women included in a community randomised trial of micronutritient supplementation aged 15–40 y, 1994–1997	Hw = 74.2Pv = 10.9	73% (<110 g/L)	Multivariate logistic regression: age, gestation, SES, Pv & serum retinol.	[48]
Yen Thanh district, Vietnam	371 women living in 6 purposively selected communities, 2003	Hw = 21.5Pf/Pv = 0	43.7% (<110 g/L)	Multivariate logistic regression: education, dietary intake, gestation & Al.	[59]

a Hw = hookworm; Pf = *Plasmodium falciparum*; Pv = *Plasmodium vivax*; Sm = *Schistosoma mansoni*; Sh = *S. haematobium*; Tt = *Trichuris trichiura*; Al = *Ascaris lumbricoides*; Hp = *Helicobacter pylori*. SES = socio-economic status.

### Effect of anthelmintic treatment

Our literature search identified two randomised controlled trials (RCTs) on the impact of anthelmintic treatment in pregnancy, two non-randomised intervention trials, and two observational studies (Table 2). All the studies showed a benefit of deworming for maternal or child health, but since a variety of outcomes measures were employed it is difficult to compare study findings quantitatively. Both RCTs had clear objectives, provided sample sizes calculations and undertook analyses adjusted for potentially confounding factors, but only the RCT in Peru [24) presented a flow of participants through each stage and baseline characteristics of each group, and stated there was adequate concealment of assignment of participants. Of the two RCTs, only the one in Sierra Leone demonstrated a statistically significant benefit of treatment, with the decline in Hb during pregnancy 6.6 g/L less in women treated with albendazole compared to untreated women [25]. The study also showed an additive impact of deworming and iron-folate supplementation, with 13.7 g/L less decline in Hb over the course of pregnancy compared to controls. The RCT in Peru found no impact of treatment on Hb or mean birthweight but showed a significant decrease in the prevalence of very low birthweight with anthelmintic treatment [24]. In the Sierra Leone RCT, no adverse pregnancy outcomes were found to be linked to albendazole. The RCT in Peru found no significant difference between the mebendazole and placebo groups in the frequency of miscarriages, malformations, stillbirths, early neonatal deaths and premature babies [24],[26].

**Table 2:** The impact of anthelmintic treatment (mebendazole (MBZ) or albendazole (ABZ)) on the health of pregnant women in the developing world.

Study	Country	Prevalence of STH^a^	Baseline prevalence of anaemia ^b^	Study design	Participants	Interventions	Results
*RCTs*							
[25]	Sierra Leone	Hw = 65% Al = 20% Tt = 74%	56.0%	Randomised placebo-controlled factorial trial of ABZ and iron-folate supplementation (n = 125). Assignment using random numbers	Women aged 15–38 y attending antenatal clinic in first trimester, Hb>g/L, gestational age <14 weeks at baseline	400 mg ABZ 36 mg ferrous gluconate plus 5 mg folic acid daily	Between 1^st^ and 3^rd^ trimester, Hb of women receiving ABZ declined 6.6 g/L less than control; the corresponding value for deworming and iron-folate supplementation was 13.7 g/L
[24]	Peru	Hw = 47% Al = 64% Tt = 82%	47.2%	Double-blind randomised placebo-controlled trial or MBZ plus iron versus placebo plus iron (n = 1042). Concealed assignment using random numbers	Women aged 18–44 y attending 12 antenatal clinics in second trimester, Hb>g/L, gestational age <18–26 weeks at baseline, and not received treatment for 6 months	500 mg MBZ 60 mg ferrous sulphate daily for 1 month	No difference in maternal anaemia or mean birthweight between groups; however, lower prevalence of very low birthweight babies in MBZ group
*Non-randomised intervention trials*							
[27]	Cote d'Ivoire	Hw = 50 Al = 78%	NA^c^	Non-randomised drug trial	Women aged 15–38 y attending antenatal clinic	500 mg Pyrantel pamoate daily for three days	Decrease in severe anaemia and 6-month infant mortality; increase in birthweight
[28]	Sri Lanka	Hw = 41.4	65.4%	Non-randomised intervention trial of iron supplementation and anthelmintics (n = 115)	Randomly selected pregnant plantation workers	Unspecified (probably MBZ) 60 mg ferrous sulphate and 0.25 mf folic acid daily for 1–2 months	Anthelmintic treatment in addition to iron supplementation improved Hb more than iron supplementation alone
*Observational studies*							
[29]	Nepal	Hw = 74% Al = 59% Tt = 5%	NA	Non-randomised community-based study investigating receipt of ABZ and health (No doses = 58; One dose = 543; Two doses = 2726)	Pregnant women previously enrolled in a cluster-randomised trial followed up 6 months post-delivery.	400 mg ABZ	Decrease in severe anaemia and 6-month infant mortality; increase in birthweight
[30]	India	NA	68.7%	Pre-post (18 months) community based evaluation (n = 828) of deworming and iron-folate supplementation.	Randomly selected pregnant women from two areas (one intervention; one control).	100 mg MBZ twice daily for three days plus 60 mg ferrous sulphate from fourth month of pregnancy	Improvement in Hb (6.4–8.4 g/L according to trimester)

Adapted and expanded from [60].

a Hw = hookworm; Al = *Ascaris lumbricoides*; Tt = *Trichuris trichiura*; *^b^* Defined as Hb<110 g/L; ^c^ Not available.

The two non-randomised intervention trials presented data on the impact of anthelmintic treatment on Hb. A study in Cote d'Ivoire included 32 pregnant women treated with pyrantel pamoate and showed that the prevalence of hookworm decreased by 93% and Hb increased by 6 g/L over the course of the pregnancy [27]. A study in Sri Lanka also showed that treatment increased Hb in pregnant women, and found that providing both mebendazole and iron supplementation had a greater impact on Hb than iron supplementation alone [28]. The observational study in Nepal compared women who had received anthelmintic treatment to those who did not, and found that treatment had significant beneficial effects on severe anaemia, birthweight and infant mortality [29]. The other observational study on pregnant women, in India, also found that co-administration of mebendazole and iron supplementation resulted in improved Hb [30].

### Burden of hookworm in pregnant women in sub-Saharan Africa (SSA)

Using GPW3.0β population estimates and country-specific age-sex structures, we estimate that in 2005 there were 148 million women of reproductive age (15–49 years) in hookworm endemic countries in SSA. Overlaying this surface with our model of hookworm prevalence we estimate that 37.7 million women of reproductive age are infected with hookworm. On the basis of number of live births occurring in SSA, we estimated that the number of pregnant women in SSA in 2005 was 25.9 million of which approximately 6.9 million were infected with hookworm.

## Discussion

That human hookworm infection results in intestinal blood loss which, in turn, can contribute to anaemia is well-established [8]. What has remained unclear and hindered public health policy and planning is the extent to which hookworm is associated with anaemia during pregnancy. The results of our systematic review quantify this relationship and confirm that heavy intensities of hookworm infection are associated with lower levels of haemoglobin than light infection intensities. This finding corroborates previous studies among school-aged children that show a relationship between infection intensity and haemoglobin [31]–[33].

Over forty years ago, Roche & Layrisse [31] in their seminal study on hookworm anaemia identified four conditions necessary to show an association between hookworm infection and Hb: a large sample size; quantitative measures of haemoglobin and hookworm infection; sufficient variation in infection levels; and few other competing causes of anaemia. These conditions are also relevant to interpreting the current results: in particular, the absence of estimates of hookworm intensity resulted in the exclusion of studies, some of which, reported no association between hookworm infection and the risk of anaemia [34]–[36]; while others reported a significant association [37]–[38]. Anaemia in developing countries has multiple causes, including micro-nutrient deficiencies, infectious diseases and inherited disorders [39], and as such, the observed relationship between Hb and hookworm infection may be confounded by other causes of anaemia. Furthermore, residual confounding may exist among studies which did not adjust for socio-economic status, which may lead to an overestimation of association. However, nine of the 13 studies undertook some form of analysis which adjusted for potential confounding variables, including dietary intake, gestation age, and co-infections (Table 1), thereby adding weight to the observed associations; only four studies adjusted for socio-economic status.

The contribution of hookworm infection to maternal anaemia is such that all women of child-bearing age could benefit from periodic treatment in hookworm endemic areas, and that women harbouring the heaviest infections are likely to benefit most. Previously, a systematic review of randomised controlled trials investigating the impact of anthelmintic treatment on haemoglobin among school-aged children concluded that treatment against intestinal nematode infections resulted in an increase in haemoglobin of 1.71 g/L (95% confidence intervals 0.70–2.73) [40]. However, there were a number of important omissions in the study, including the failure to distinguished between different helminth species or account for intensity of infection, which may have under-estimated the true treatment effect [41]. The treatment studies among pregnant women reported here found that albendazole was effective in reducing the decline in haemoglobin that typically occurs during pregnancy [25], but that the effect was less apparent with mebendazole [24]. This may reflect the lower efficacy of mebendazole versus albendazole in treating hookworm infection [42],[43]. However, there is a trade-off between efficacy and safety since mebendazole is poorly absorbed from the gut whereas albendazole is turned into a sulfoxide metabolite that gets widely distributed in the tissues. In addition to drugs used, there are other potential reasons accounting for the difference in the observed impact of anthelmintic treatment on haemoglobin. These include higher intensities of hookworm among women in Peru than among the women in the Sierra Leone study. In addition, different underlying aetiologies of anaemia may be relevant, such differences in iron deficiency anaemia and malaria and schistosome transmission intensity [39]. Finally, although we did not quantitatively assess the quality of the studies, reporting of the RCT in Sierra Leone was incomplete and it is possible that there were methodological differences that were associated with observed treatment effects [14].

Despite the potential benefits of anthelmintic treatment during pregnancy, few countries have included deworming in their routine antenatal care (ANC) programmes, with only Madagascar, Nepal and Sri Lanka doing so routinely. It is suggested that a fear of adverse birth outcomes as well as a lack of safety data, especially country-specific data, represents a barrier for many ministries of health including anthelmintics into their ANC programmes. The evidence from the RCTs included in this review found no evidence of an increased risk of adverse events following treatement. This is consistent with other observational studies which have investigated the safety of mebendazole in pregnant women (for a recent review of studies, see [26]). We feel that the findings of the present paper make clear that hookworm in pregnancy is prevalent and important, and we strongly encourage that a substantial review of the safety evidence is undertaken, perhaps by WHO and its partners.

The finding that co-administration of deworming and iron supplements has a greater impact on haemoglobin than deworming alone supports the assertion that deworming is unlikely to replenish iron stores in the short term, and needs to be combined with iron supplementation, particularly among populations whose diets is low in bioavailable iron [10]. In addition, a review of the impact of malaria-related anaemia among pregnant women in sub-Saharan Africa suggested that over a quarter of cases of severe anaemia were attributable to malaria [44], while other evidence shows that anaemia burden can be reduced effectively by anti-malarial intermittent preventive treatment (IPT) [7]. An effective package to improve maternal anaemia should therefore ideally include IPT, iron supplementation and anthelmintic treatment. Interestingly, a recent case control study of the causation of severe anaemia in young children in Malawi also concludes that hookworm has tended to be overlooked as a causal factor [45]. The value of combining deworming with micronutrient supplementation for children has previously been emphasized [46].

We found only slight evidence of publication bias, and this is likely to be less important than the numerous other factors that may introduce heterogeneity [17], such as transmission of malaria and schistosomiasis, iron and nutritional intake, diagnostic accuracy in quantifying Hb and hookworm intensity. Furthermore, hookworm species may be important but in the reported studies, no distinction was made between *N. americanus* and *A. duodenale* because of the practical difficulties of differential diagnosis. Pathological studies indicate that *A. duodenale* causes greater blood loss than *N. americanus* [47], with epidemiological studies among Zanzibari schoolchildren suggesting that *A. duodenale* is associated with an increased risk of anaemia [48]. Thus, where hookworm is exclusively *A.duodenale*, such as in Nepal [49], the observed effect on maternal anaemia might be greater.

In 1995, Bundy and colleagues estimated that in low income countries, 44 million (35.5%) out of 124 million pregnant women were infected with hookworm [9]. Here we estimate that 6.9 million (26.7%) out of 25.9 million pregnant women in SSA are infected with hookworm. Our current estimates are more precise since they are the first to explicitly include the fine spatial variation in distribution of both infection and population. They suggest that the earlier methodology may have overestimated the proportion of pregnant women infected. On the other hand, the reliance on infection prevalence data from surveys of schoolchildren, in the absence of data from adult women, means that both estimation procedures are likely to result in under-estimates. Nonetheless, the estimates suggest that between a quarter and a third of pregnant women in sub-Saharan Africa are infected with hookworm and therefore at risk of preventable hookworm-related anaemia.

In conclusion, this systematic review presents evidence that increasing hookworm infection intensity is associated with lower haemoglobin levels in pregnant women in poor countries. The chronic and recurring nature of hookworm infection throughout the reproductive years means that it may have a chronic impact on the iron status of infected women, potentially contributing to their morbidity and mortality and that of their children. In many developing countries it is policy that pregnant women receive anthelmintic treatment but in practice coverage rates are often unacceptably low. We suggest that efforts are made to increase the coverage of anthelmintic treatment and iron supplementation, with, where appropriate, intermittent preventive treatment for malaria.
